# Bedside adipose tissue metabolism in acute critical care illness monitored by microdialysis (MD)

**DOI:** 10.1186/2197-425X-3-S1-A582

**Published:** 2015-10-01

**Authors:** M Theodorakopoulou, F Frantzeskaki, S Apollonatou, N Nikitas, P Kopterides, A Diamantakis, I Dimopoulou

**Affiliations:** University Hospital Attikon, 2nd Critical Care Department, Athens, Greece

## Introduction

MD is a bedside *in vivo* sampling technique that permits continuous analysis of a patient's interstitial fluid chemistry. As the interstitial fluid bathes the cells, its composition reflects the local metabolic activity of those cells thus reflecting intracellular metabolic changes and disorders. The primary substances that provide information about metabolism are lactate, pyruvate, glycerol and glucose. In addition, blood metabolic alterations have been well characterized in severely ill patients. In vivo MD is performed by implanting a commercially available catheter that mimics a blood capillary at the site of interest.

## Objectives

In this study, we used MD to assess adipose tissue metabolic patterns in a large number of ICU patients.

## Methods

Upon admission in the ICU, a microdialysis catheter (CMA60, CMA, Solna, Sweden) was inserted into the subcutaneous adipose tissue of the upper thigh. Dialysate samples were collected in microvials and were analyzed immediately for glucose, pyruvate, lactate, and glycerol using a mobile CMA ISCUS analyzer. The lactate/pyruvate (L/P) ratio was automatically calculated. Measurements were performed 6 times per day for 3 consecutive days. The daily mean values of MD measurements were calculated for each patient. Glycolysis was defined as LP ratio < 30 and tissue lactate>2 mmol/L, lipolysis was defined as tissue glycerol>200 mmol/L, ischemia was defined as LP ratio>30 and pyruvate level < 70 mmol, while mitochondrial dysfunction was defined as LP ratio>30 and pyruvate>70 mmol.

## Results

The study included 203 mechanically ventilated medical, trauma and surgical patients aged>18 yrs. Median age was 67 years. All septic patients met the ACCP/SCCM criteria for sepsis. Apache II and SOFA scores were calculated upon admission. Sepsis stages included: SIRS n = 24, severe sepsis n = 46 and septic shock n = 133. It is wel known that critical care sepsis is characterized by an excessive release of extracellular glucose, lactate, and glycerol. Septic patients had higher glycerol, glucose and lactate levels but kept lower pyruvate and L/P ratio levels thus mirroring the well-known sepsis related blood metabolic alterations. Lactate and glycerol were higher in mitochondrial dysfunction, glucose was lower in ischemia and L/P ratio was higher in mitochondrial dysfunction and ischemia. Only 19 patients (9%) had a normal metabolic profile. Lipolysis and glycolysis were present in 73% and 52% of the patients respectively. Mitochondrial dysfunction was observed in 26 patients (13%) and ischemia in 27 patients (13%). ICU mortality was 53%. The mortality observed in the study population was 77% in mitochondrial dysfunction, 52% in ischemia and 49% in patients that exhibited neither mitochondrial dysfunction nor ischemia.

## Conclusions

Bedside subcutaneous adipose tissue MD is possible to diagnose and separate diverse metabolic patterns in ICU patients. Lipolysis is the most common observed metabolic pattern followed by glycolysis.

